# Highly Elastically Deformable Coiled CNT/Polymer Fibers for Wearable Strain Sensors and Stretchable Supercapacitors

**DOI:** 10.3390/s23042359

**Published:** 2023-02-20

**Authors:** Jin Hyeong Choi, Jun Ho Noh, Changsoon Choi

**Affiliations:** 1 Department of Energy and Materials Engineering, Dongguk University, 30 Pildong-ro, 1-gil, Jung-gu, Seoul 04620, Republic of Korea; 2 Department of Advanced Battery Convergence Engineering, Dongguk University, 30 Pildong-ro, 1-gil, Jung-gu, Seoul 04620, Republic of Korea; 3 Research Center for Photoenergy Harvesting & Conversion Technology (phct), Dongguk University, 30 Pil-dong-ro, 1-gil, Jung-gu, Seoul 04620, Republic of Korea

**Keywords:** polymer composite fibers, coil structure, carbon nanotubes, strain sensor, supercapacitor

## Abstract

Stretchable yarn/fiber electronics with conductive features are optimal components for different wearable devices. This paper presents the construction of coil structure-based carbon nanotube (CNT)/polymer fibers with adjustable piezoresistivity. The composite unit fiber is prepared by wrapping a conductive carbon CNT sheath onto an elastic spandex core. Owing to the helical coil structure, the resultant CNT/polymer composite fibers are highly stretchable (up to approximately 300%) without a noticeable electrical breakdown. More specifically, based on the difference in the coil index (which is the ratio of the coil diameter to the diameter of the fiber within the coil) according to the polymeric core fiber (spandex or nylon), the composite fiber can be used for two different applications (i.e., as strain sensors or supercapacitors), which are presented in this paper. The coiled CNT/spandex composite fiber sensor responds sensitively to tensile strain. The coiled CNT/nylon composite fiber can be employed as an elastic supercapacitor with excellent capacitance retention at 300% strain.

## 1. Introduction

A paradigm shift from portable devices to wearable devices during the fourth industrial revolution has sparked tremendous research interests in developing one-dimensional (1-D) fiber-based stretchable conductors [1,2,3,4,5,6]. As effective building blocks for various wearable applications, these devices are continuously exposed to mechanical deformations originating from the curvature shapes and frequent movements of the human body. Therefore, it is of significant importance to achieve high stretchability without significant performance loss for both user convenience and device reliability. Particularly, the piezoresistive sensitivity (the fractional change in resistance under applied strain), known as the gauge factor (GF), can become a crucial criterion for the two possible applications of fiber conductors: strain sensors and stretchable electrodes. For example, a fiber conductor with a high piezoresistive effect is expected to be suitable for a strain sensor [7,8] because it can induce a large electrical change against the applied strain. On the other hand, fiber conductors with insensitive piezoresistivity may act as stretchable electrodes [4,5,6,9,10] for resistance-stable devices, such as artificial muscles [11,12], electronic interconnects [13,14,15], supercapacitors, and batteries [16,17,18,19].

Fibrous strain sensors are developed by incorporating conductive filers (e.g., silver nanoparticles/wires and carbon nanomaterials) [7,20,21,22] into the elastomeric substrates (e.g., Ecoflex, polydimethylsiloxane, and polyurethane) [2,3] through various spinning methods. The sparse percolation network of conductive fillers proportionally increases their electrical resistance upon elongation of the composite fibers, providing a working mechanism for the piezoresistive type sensor. Unfortunately, there is a trade-off between the available strain range and the piezoresistive effect; hence, improving electrical response by minimizing the loading density of the conductive fillers inevitably leads to a limited sensing range. Meanwhile, stretchable electrodes are commonly required to be able to extend significantly with negligible resistance changes (i.e., low piezoresistivity). In this regard, coil-structured conductors have recently been proposed as promising candidates for stretchable electrodes due to their reversible deformability, wide range of geometries, and wearability [6,23]. The fundamental problem is that the decrease in contact area between helical loops causes a longer electrical pathway in the longitudinal direction of the fiber [24] which may hinder the achievement of reliable performance of the stretchable devices. Therefore, if one can systematically control the piezoresistive effect of fiber conductors in a simple way while roughly maintaining their stretchability, it may be possible to widen its applicability to both sensors and stretchable electrodes.

Here, we propose a facile, simple, and scalable method for achieving both high elasticity and a tunable strain-dependent electrical response for dual-functional potentials, such as strain sensors and extensible supercapacitor electrodes. In this study, we adopted a sheath-core structural fiber composed of an elastic polymer core and conductive carbon nanotube sheaths. In particular, a control of coil index (the ratio of average coil diameter to fiber diameter) on the coil architecture can change its electromechanical characteristics by inducing a morphological difference; this is a critical variable for designing a strain sensor and stretchable electrode. In addition, with the combined effect of elastic polymer core (Spandex or Nylon) and helical coil geometry, the coiled carbon nanotube (CNT)/polymer fiber can exhibit high tensile properties (300%). By inducing the difference in the coil index-dependent piezoresistive response of the fiber with tensile strain, the presented coiled CNT/polymer fiber can operate as a strain sensor and a stretchable electrode of a supercapacitor. The coiled CNT/spandex composite fiber exhibits sensitive resistance responses to tensile deformations due to the continuous coil openings of the fibers. Furthermore, with no significant electrical change under the applied strain (over 10%), the coiled CNT/Nylon composite fiber is developed into a stretchable supercapacitor, performing a large specific capacitance, superior rate performance, and cycle stability.

## 2. Results and Discussion

### 2.1. Morphological Information and Electron Transport in Coiled CNT/Polymer Fibers

**Figure 1:**
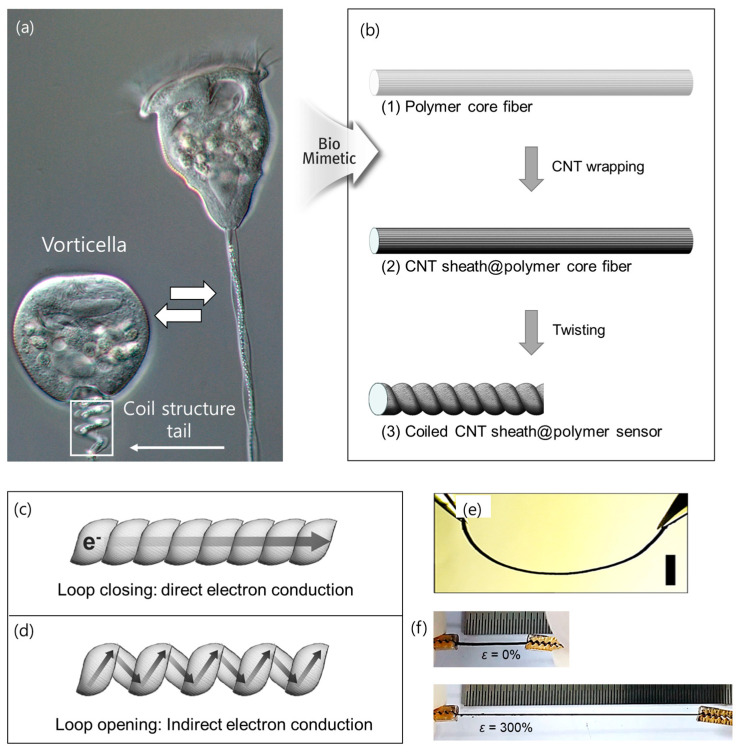
Morphology and electron transfer mechanism for the coiled carbon nanotube (CNT)/polymer fiber (**a**) Photograph showing Vorticella before (**left**) and after body stretching (**right**) via the loop opening of the coil-structured tail. (**b**) Schematic of biomimetic strategy for the fabrication of coil-structured CNT/polymer fibers. (1 and 2) The CNT sheath is being wrapped around the core fiber; (3) the fiber is twisted many times to coil the fiber. The electron transfer mechanisms in the coil fiber-based sensor are compared: (**c**) (initial state) direct electron transfer through the closed loops, and (**d**) (stretched state) indirect electron transfer along the coil helix through the opened loops. (**e**) Photograph shows the bending of coiled CNT/polymer fiber sensor with tweezers. (**f**) Photographs of coiled CNT/polymer fiber sensor (initial length: 1 cm) before (**above**) and after (**below**) 300% strain application (final length: 4 cm).

Vorticella is one example of this coil structure which can be found in nature. It can extend its body length from 30–100 μm to 100–200 μm by opening the coil-structured tail, as shown in Figure 1a. To bio-mimic the high stretchability of Vorticella, we fabricated coil-structured CNT/polymer composite fibers (Figure 1b). The precursor fiber was prepared by covering the core polymer fibers into a CNT sheet (Figure S1), which was mechanically drawn from a vertically aligned multi-walled CNT forest (as reported in [25]). The CNT sheath and core polymer function as an electrically conductive pathway and tough substrate, respectively. The structural properties and elemental constituents of the used CNT sheets are presented in Figure S2. As shown in Figure S2a, the XRD patterns displayed two distinguishable diffraction peaks: the strong C (002) peak at approximately 26 represents the graphitic characteristic of CNTs, and the peak at approximately 43 is indexed to the (100) planes of the nanotube structure. These XRD patterns clearly confirm the crystallinity of the CNTs used in this work, which aligns favorably with previous studies [26]. Meanwhile, the Raman spectra exhibited two main bands (D and G) at 1350 and 1585/cm (Figure S2b) which are associated with the presence of disorders in the sp2 structure, and the stretching of the C–C bond (sp^2^), respectively. The intensity ratio (ID/IG) between these two bands, representing the relative content of defects in the graphitic carbon structure, was calculated as 0.21. This low D band intensity of the used CNTs indicates their relative purity. In addition, the X-ray photoelectron spectroscopy (XPS) spectra also showed a distinct carbon (284 eV) peak with a negligible intensity oxygen (533 eV) peak (Figure S2c), supporting the above Raman results.

Although the pristine CNT yarn can be fully coiled without the help of a polymeric substrate, its stretchability is limited mostly to values below 100% strain owing to the defects of the CNT [27]. To improve the stretchability, we adopted strong and commercially available core polymers with 200 µm diameters (nylon or spandex) (Figure S3). Subsequently, ethanol was dropped onto the composite fiber surface to create surface-tension-based densification during ethanol evaporation. The resulting CNTs adhere strongly to the core polymers without noticeable delamination. The precursor CNT/polymer fibers can be transformed into coils by twisting them many times. First, the precursor fiber was twisted at approximately 2400 turns/m (relative to the initial fiber length), then it was twisted at 4000 turns/m. The final coil was 66% shorter. The working mechanism of the presented coiled fiber sensor is based on a conductivity decrease due to the progressive elimination of inter-coil contact when the sensor is deformed in the tensile direction. Losing the inter-coil contact forces electrons to loop along the coils, thereby traveling between adjacent contacting coils, which results in a roughly linear relationship between the strain and resistance. This origin of the resistance changes versus the applied strain is schematically illustrated in Figure 1c,d, respectively. As the coil structure of our sensors provides elastomeric deformability, the sensor can be bent (Figure 1e) and even stretched to 300% extension. The photographs in Figure 1f show the 1 cm long CNT/polymer fiber sensor, initially coiled, and stretched to 4 cm without noticeable residual deformation.

### 2.2. Electrical and Mechanical Responses with Coil Loop Opening Properties

**Figure 2:**
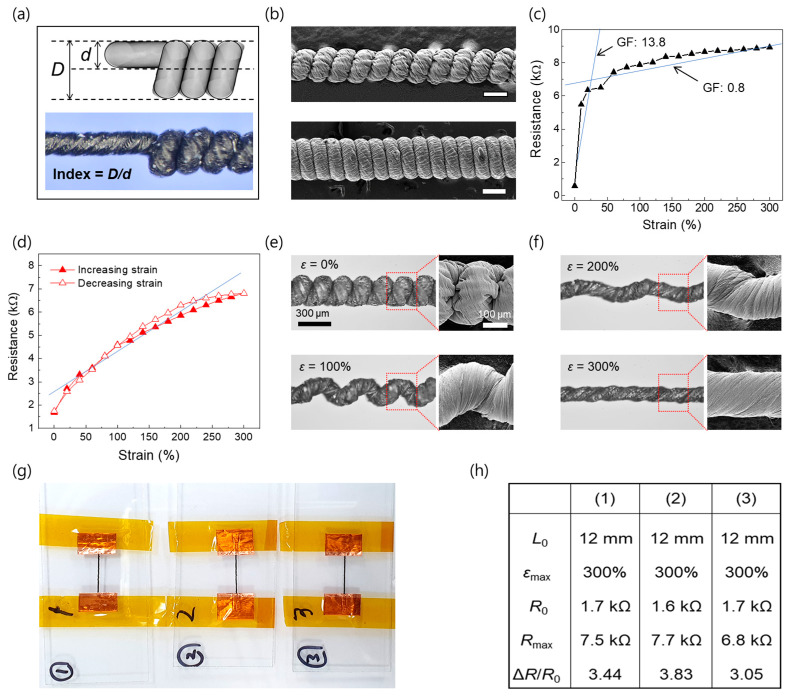
Electromechanical response and coil loop opening characteristics (**a**) Schematic (**above**) and photograph (**below**) of the definition of the coil index, which can be calculated by dividing the average diameter of the coil fiber (*D*) by the diameter of the precursor fiber (*d*). (**b**) Scanning electron microscopy (SEM) images of coiled CNT/polymer fiber sensors with low (**above**: scale bar = 200 µm) and high indexes (**below**: scale bar = 300 µm). Resistance changes versus strain of coiled CNT/polymer fiber with (**c**) high (2.9) and (**d**) low index (1.9). The slope of the blue lines represents the piezoresistive sensitivity (i.e., GF). Photographs and magnified SEM images show progressive coil loop opening at (**e**) 0% and 100%, and surface buckles unfolding at (**f**) 200% and 300% strains. (**g**) Photograph of three coiled CNT/polymer fiber sensors (length: 1.2 cm). The table in (**h**) lists the performance characteristics of the three fiber sensors.

Moreover, the conductivity decrease of the coiled CNT/polymer fiber sensors can be controlled based on their coil index. As presented in Figure 2a, the coil index can be calculated by dividing the average diameter of the coil fiber (*D*) by the diameter of the precursor fiber (*d*). The coil index can be altered via the applied load during twist insertion [28]. In addition, in this study, we selectively assume the other parameter, i.e., the elasticity of the core polymer fiber. For example, the coil index of the sensor with a spandex fiber (highly elastic) as the core fiber is relatively low (approximately 1.9), whereas the index of the nylon fiber (weakly elastic) is high (up to approximately 2.9). It seems that the highly elastic core fiber has a lower coil index because it can also be squeezed, which increases the coil bias angle. Figure 2c shows the resistance change versus the strain of the coiled CNT/nylon fiber with 2.9 coil index. The GFs of the presented sensors are largely divided into two values with respect to the strain: high GFs of approximately 13.8 in the low strain range (from 0% to 50%), and low GFs of 0.8 in the high strain range (up to 300%).

Whereas the resistance abruptly increases with increasing strain in the low strain range (from 0.19 kΩ/cm at 0% strain to 1.83 kΩ/cm at a strain below 10%), the sensors experience only slight changes in their electrical resistance from approximately 10% to 300% strain. However, the coiled CNT/spandex fiber sensors with the low coil index exhibit a greatly improved sensing ability; the roughly constant sensitivity (GF = 1.15) and linear resistance change with respect to the strain are evident up to 300%; there is no noticeable hysteresis during the strain application and recovery cycles (Figure 2d). Evidently, the high coil index provides a larger coil contact area, and larger resistance changes tend to be derived at the early strain stage. Conversely, the low coil index provides a small resistance change; however, the sensing range is much larger. Another important factor that should be discussed is the elasticity of the core polymer fiber. The linear coil density (which can be defined as *N/L*, where *N* is the total number of coils for a given fiber length (*L*)) is approximately 50 coils/cm for CNT/spandex fiber sensors. Figure 2e,f shows the optical images for the separation between coil loops during fiber stretching alongside a magnified SEM image. In a macroscopic view, the coil opening process of the fiber occurred predominantly at the initial strain region (0–100%) implying an increase in the initial resistance by progressive elimination of inter-coil contact. This visible uniform and simultaneous coil loop opening decrease the coil density from 50 at 0% strain to approximately 0 coils/cm at 300% strain. On the other hand, in a microscopic view, it was shown that the surface micro-buckles were mainly unfolded rather than the opening of coil loops in the region higher than 100% strain. To investigate the reproducibility of the coiled CNT/polymer fiber sensors, three coiled CNT/spandex fiber sensors were prepared (Figure 2g); the corresponding GFs are compared for up to 300% strain in Figure 2h. The specimens performed similarly within the standard deviation, thereby proving the reproducibility of the presented fiber sensors.

The large change in the resistance per fiber length (5.08 kΩ/cm) up to 300% strain is remarkable; it probably results from the reversible progressive coil loop opening process with increasing strain. The resulting large sensing range is comparable to those of previously reported sensors [8,29,30].

### 2.3. Torque-Stable and Weavable Coiled Fiber Sensors with Styrene Ethylene Butylene Styrene (SEBS) Passivation

**Figure 3:**
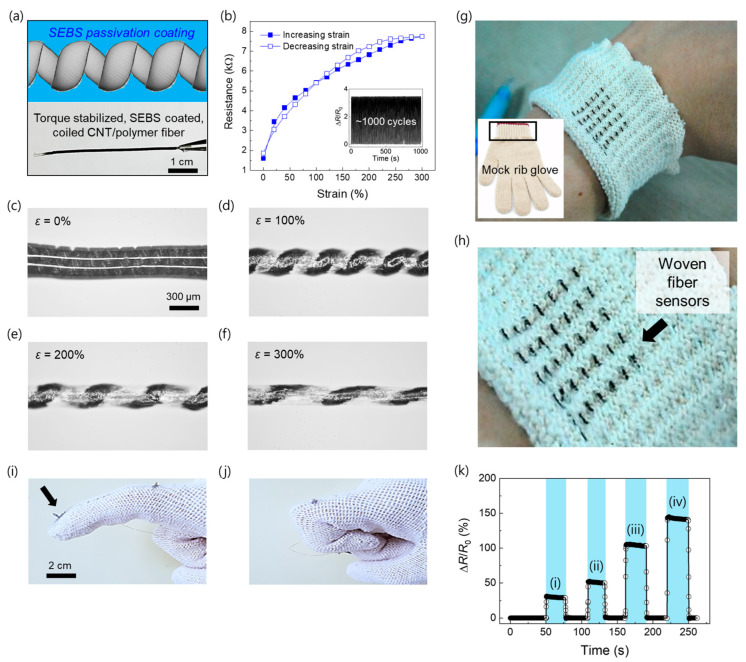
Demonstration of torque stability and weavability of the coiled fiber sensors for wearable applications. (**a**) Schematic shows SEBS package-coated coiled CNT/polymer fiber sensor, and (**b**) its resistance with respect to tensile strain. The inset of (**b**) shows the resistance change ratio during 1000 repeated loading/unloading with 300% strain. Photographs show the SEBS package-coated, coiled CNT/spandex fiber sensor at different strain values: (**c**) 0%, (**d**) 100%, (**e**) 200%, and (**f**) 300%. The photographs in (**g**) present seven coiled CNT/spandex fiber sensors woven into the wristband part of a mock ribbed glove to replace the mock rib fibers; (**h**) shows the magnified image. Photographs present finger movements (**i**) before and (**j**) after bending and (**k**) corresponding resistance change versus time at different finger bending angles ((i) 25°; (ii) 50°; (iii) 75°; and (iv) 100°).

In general, the twist insertion-based coiling method introduces torque instability in the yarns owing to the severe torsional stress that is introduced during coiling. Therefore, coiled yarns tend to untwist vigorously or snarl when they are untethered [31,32,33]; torque instability is the main limitation that restricts the widespread application of coiled yarns. Although the coil structure can be conserved by tethering the two ends of the yarns, the additional tethering gadgets increase the volume and weight of the total system, thereby significantly degrading the volumetric and gravimetric performance characteristics, respectively. To prepare coiled CNT/polymer fiber sensors with torque stability, we deposited a styrene ethylene butylene styrene (SEBS) packaging layer onto the coiled CNT/polymer fiber, as shown in Figure 3a. The SEBS packaging layer effectively stabilizes the coil structure. Thus, the SEBS-coated coiled CNT/polymer fiber can remain in the free-standing state without tethering gadgets, as shown in the photograph in Figure 3a. The coating has a negligible effect on the electron transfer in the coiled CNT/polymer fibers. This is confirmed by the resistance–strain curve in Figure 3b. The plot and corresponding GF of the SEBS-coated coiled CNT/polymer fiber are almost identical to those of the bare coiled CNT/polymer fibers. Additionally, the fiber sensors showed stable sensing performance during 1000 repeated strain (~300%) application cycles with a rate of one cycle of stretch/release per second (inset of Figure 3b). We observed the progressive and uniform coil loop opening process of the SEBS-coated coiled sensors during the application of up to 300% strain (Figure 3c–f). The coiled CNT/polymer fiber is flexible, stretchable, and sufficiently strong to be woven into a textile. Although a coiled CNT/nylon fiber may be more favorable for sensitive strain sensing due to its high coil index, we employed a coiled CNT/spandex fiber with mechanical softness as a wearable sensor for practical applications. Therefore, these coiled CNT/spandex fiber sensors are powerful candidates for wearable devices. We confirmed this by weaving the coiled sensor fibers into mock rib-structured gloves. More specifically, the coiled CNT/polymer fibers were woven into the wrist (Figure 3g,h) and finger (Figure 3i,j) parts to replace the original fibers. To assess the applicability of the wearable glove sensors for the measurement of finger movements, a 4 cm coiled CNT/polymer fiber was sewn into the finger joint region of a commercially available glove; the bending angles of the knuckle during the finger motions were monitored by measuring the real-time resistance changes (Figure 3k). The bending degrees of the finger were 25°, 50°, 75°, and 100°; the corresponding resistance changes of the woven coiled CNT/polymer fiber are roughly proportional with GFs of 1.15.

### 2.4. Coiled CNT/Polymer Fiber Electrodes for Elastic Supercapacitors

**Figure 4:**
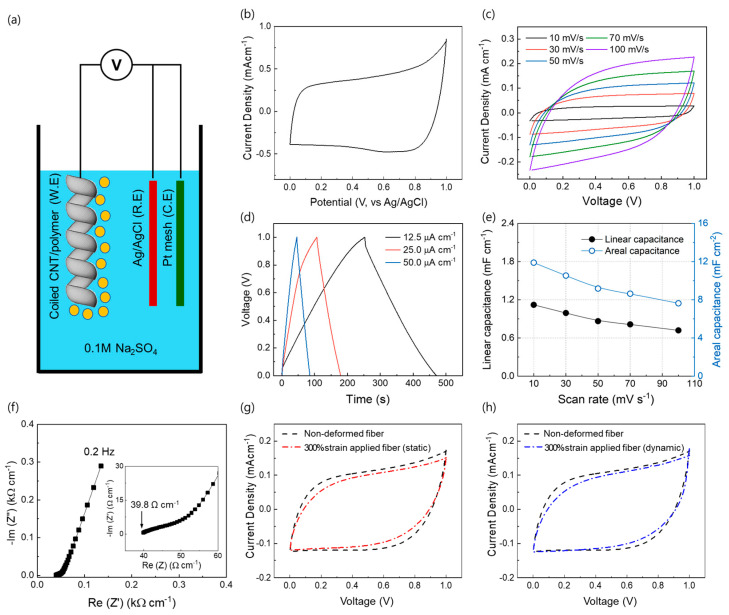
Electrochemical double layer (EDL) charge storage performance of coiled CNT/polymer fiber and capacitance retention during fiber stretching. (**a**) Schematic of the three-electrode system for measurement of the capacitance of coiled CNT/polymer fiber sensor (i.e., the working electrode); Ag/AgCl and Pt mesh were used as reference and counter electrodes in 0.1M Na_2_SO_4_ electrolyte, respectively. (**b**) Measured cyclic voltammogram (CV) curve (at 300 mV/s) of the three-electrode system. (**c**) CV curves of coiled CNT/polymer fiber supercapacitor measured at 10–100 mV s^−1^. (**d**) Galvanostatic charge–discharge curves measured from 12.5 µA cm^−2^ to 50.0 µA cm^−2^ current densities. (**e**) Calculated linear and areal-specific capacitance as a function of voltage scan rate. (**f**) Nyquist curve for the frequency range from 0.2 to 100 kHz (the inset shows the high-frequency region). The CV curves of the undeformed coiled CNT/polymer fiber are compared with the CV curves measured at a (**g**) statically and (**h**) dynamically applied strain (up to 300%).

Furthermore, coiled CNT/polymer fibers can be effective electrodes for highly deformable electrochemical double layer (EDL)-based capacitors because the CNT sheath has an ultra-large specific surface area and outstanding chemical stability [34,35]. To measure the sole characteristics of the coiled CNT/polymer fiber (i.e., the working electrode), the three-electrode system was adopted; Ag/AgCl and Pt mesh were used as the reference and counter electrodes in 0.1 M Na_2_SO_4_ (Figure 4a), respectively. The CV curves (of the three-electrode system) were recorded at 300 mV/s (Figure 4b). CV curves measured for different scan rates (from 10 to 100 mV s^−1^) and GCD curves with current densities of 12.5–50.0 µA cm^−1^ are plotted in Figure 4c,d, respectively. The rectangular CV curves (without any Faradic redox peaks) and triangle-shaped charge-discharge curves are in favorable agreement with an energy storage process by an electrochemical double-layer capacitance (EDLC) of the CNTs [36,37]. The specific capacitances of the coiled fiber supercapacitor (based on the length or area of a single electrode) are shown in Figure 4e. The calculated maximum values of the linear and areal capacitances are 1.12 mF cm^−1^ and 11.89 mF cm^−2^, respectively, at 10 mV s^−1^ scan rate. From electrochemical impedance spectroscopy (EIS) measurements in the frequency range of 0.2 Hz to 100 kHz (Figure 4f), the fiber supercapacitor exhibited the equilibrium series resistance (ESR) of 39.8 Ω cm^−1^, indicated by the intersection of the curve with the X-axis. The static and dynamic (15%/s) tensile strains (300%) resulted in negligible fluctuations in the measured CV curves (Figure 4g,h). The fiber supercapacitor also showed stable electrochemical performance with 94.2% capacity retention after 100 times full stretching (ε = 300%) cycles (Figure S4).

## 3. Materials and Methods

### 3.1. Preparation of the Coiled CNT/Polymer Composite Fibers

A 200 μm diameter commercially available Spandex (Hyosung, Seoul, Republic of Korea) and Nylon 6,6 (Coats and Clark D674 mil) were used as the polymeric core for stretchable coiled fiber. For conductive sheath, aerogel CNT sheets were drawn from a 320 μm height CNT forest (A-Tech System Co., Hwaseong-si, Republic of Korea). After wrapping the CNT sheath on the polymeric core, the precursor fiber was twisted for coiling using the customized twisting machines. As an electrolyte for electrochemical performance characterization, 1.42 g of Na_2_SO_4_ (Sigma-Aldrich, St. Louis, MO, USA) was prepared in 100 mL of deionized water and heated at 85 °C. For coiled fiber packaging, SEBS solution was prepared by dissolving SEBS particles in cyclohexane (Sigma-Aldrich, St. Louis, MO, USA) and was spray coated onto the coiled fiber.

### 3.2. Characterization

Optical images and SEM images were obtained by using an optical camera (D750, Nikon, Japan) and SEM (S-4600, Hitachi, Tokyo, Japan), respectively. X-ray photoelectron spectroscopy (XPS) measurements were conducted on an ESCALAB 250XI (Thermo Scientific, Waltham, MA, USA). X-ray diffraction (XRD) results were obtained with an X-ray diffractometer (Empyrean, 60 kV) using Cu Kα radiation (λ = 1.5405 Å). While both ends of the coiled fiber are fixed and mounted onto digital Vernier calipers (Mitutoyo, Kawasaki, Japan), The electrical measurements were performed using multimeter probes (Fluke). The electrochemical measurements used an electrochemical analyzer (Vertex EIS, Ivium Soft 4.1100).

### 3.3. Calculation

The areal capacitance of the coiled CNT/polymer fiber was calculated as follows:

$$C_{A}\left({F\;\mathrm{cm}}^{-2}\right)=\frac{I}{dV/dt}\times \frac{1}{A}$$

where I is the average discharge current, and the dV/dt is the scan rate. The A is the surface area of the coiled CNT/polymer fiber. The linear capacitance was also calculated through dividing the capacitance by the length of the initial coiled fiber.

## 4. Conclusions

We prepared a helically organized CNT/polymer composite fiber with high stretchability and controllable electrical characteristics. Owing to the coil geometry and its scalable index, the coiled CNT/polymer fiber can be used for stretchable strain sensors and supercapacitors, depending on the polymer substrate (spandex or nylon). The coiled CNT/spandex fiber sensors showed good piezoresistive responses to tensile deformations. The coiled CNT/nylon fiber was used to prepare elastic supercapacitors with reliable conduction. In summary, our manufacturing strategy for stretchable fibrous electronic devices with high elasticity and good electrical performance characteristics is suitable for the development of diverse wearable electronics.

## Data Availability

Not applicable.

## Supplementary Materials

The following supporting information can be downloaded at: https://www.mdpi.com/article/10.3390/s23042359/s1.
