# Supplementary material for: Highly Elastically Deformable Coiled CNT/Polymer Fibers for Wearable Strain Sensors and Stretchable Supercapacitors
Source: Sensors (Basel). 2023 Feb 20;23(4):2359. doi: 10.3390/s23042359 (PMC9964788; doi:10.3390/s23042359)
Supplement: Supplementary file 1 [file sensors-23-02359-s001.zip › sensors-2027133-supplementary.pdf]

Supplementary Information

Highly Elastically Deformable Coiled CNT/Polymer Fibers for Wearable Strain Sensors and Stretchable Supercapacitors

Jin Hyeong Choi [1], Jun Ho Noh [1,2] and Changsoon Choi [1,3,*]

[1] Department of Energy and Materials Engineering, Dongguk University, 30 Pildong-ro, 1-gil, Jung-gu,
Seoul 04620, Republic of Korea; locomotive97@naver.com (J.H.C.);
shwnsgh15@dongguk.edu (J.H.N.)

[2] Department of Advanced Battery Convergence Engineering, Dongguk University, 30 Pildong-ro, 1-gil, Jung-gu, Seoul 04620, Republic of Korea

[3] Research Center for Photoenergy Harvesting & Conversion Technology (phct), Dongguk University,
30 Pil-dong-ro, 1-gil, Jung-gu, Seoul 04620, Republic of Korea

* Correspondence: cschoi84@dongguk.edu

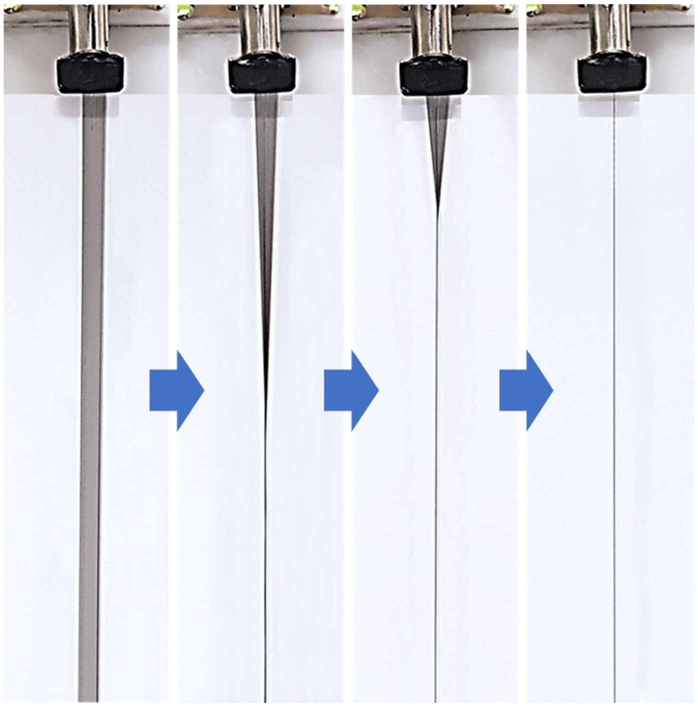

Figure S1. Photographs showing CNT sheets wrapping process on the polymer core substate.

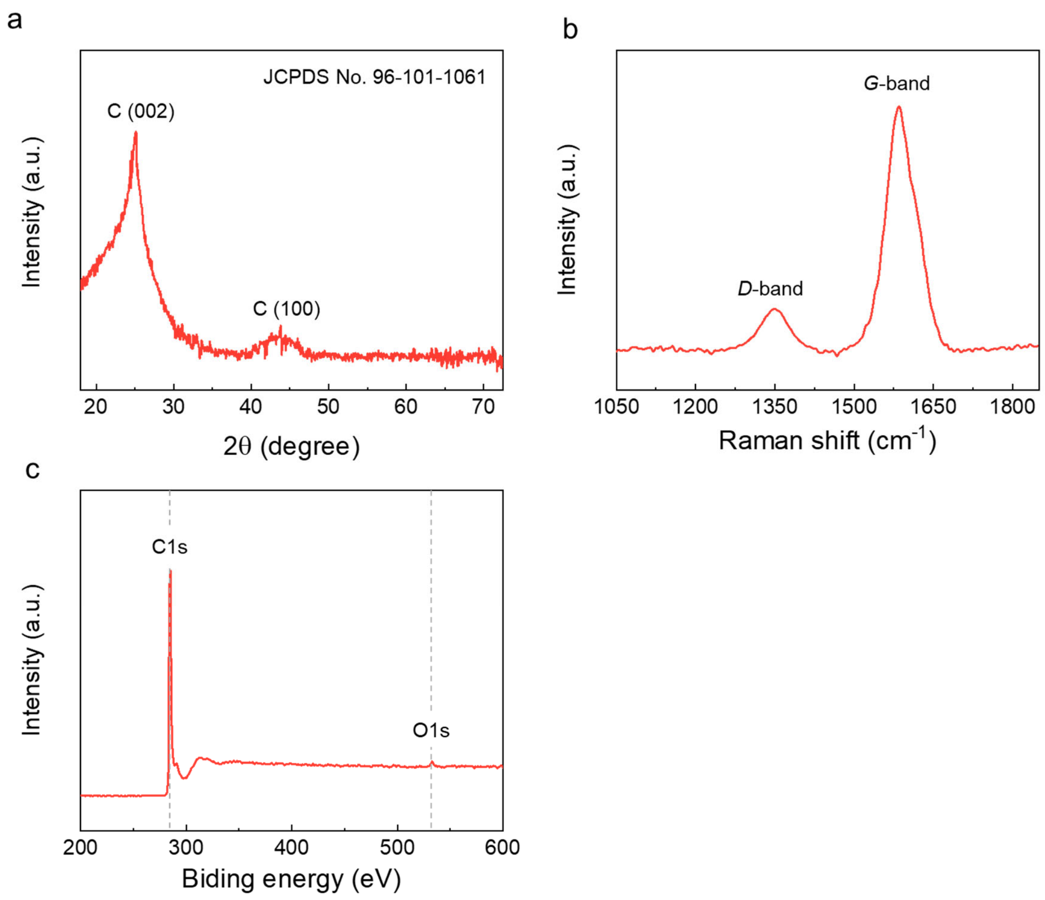

Figure S2. (**a**) XRD pattern, (**b**) Raman spectra, and (**c**) XPS spectra of the CNT sheets.

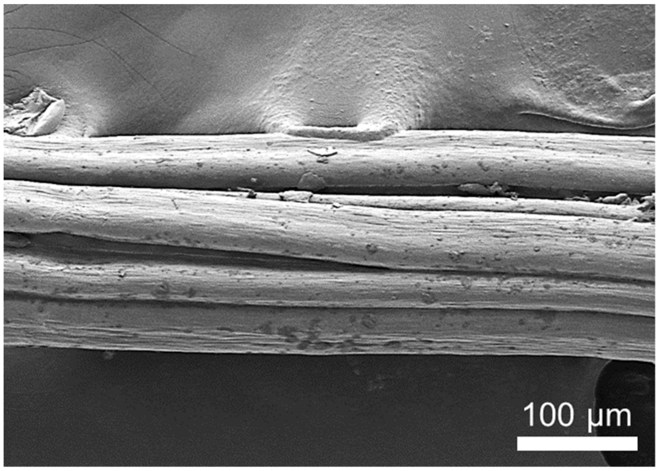

Figure S3. SEM image of the polymer (spandex) core substate.

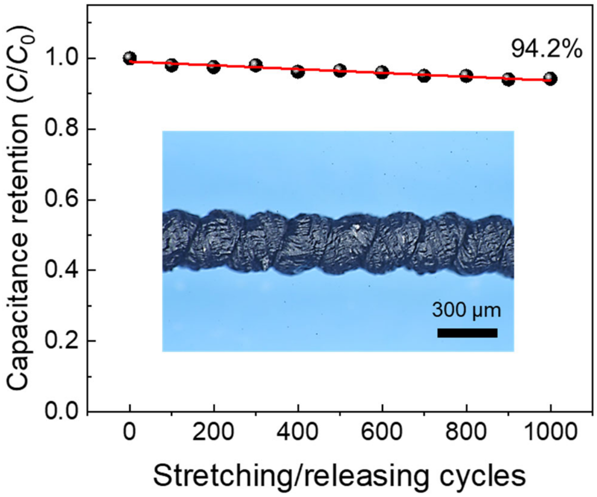

Figure S4. Capacitance retention versus stretching cycles (the inset shows an optical image of a coiled CNT/polymer fiber electrode without noticeable delamination even after immersion in an aqueous electrolyte).
